# Family-based therapy for eating disorders: from the Milan model to contemporary evidence

**DOI:** 10.3389/frcha.2026.1765146

**Published:** 2026-02-06

**Authors:** Johan Vanderlinden

**Affiliations:** Faculty of Psychology and Educational Sciences, KU Leuven University, Leuven, Belgium

**Keywords:** adolescents, anorexia nervosa, eating disorders, evidence-based practice, family-based treatment, multi-family therapy

## Abstract

Family-oriented therapy has profoundly influenced the conceptualization and treatment of eating disorders over the past five decades. Beginning with systemic pioneers such as Mara Selvini Palazzoli and Salvador Minuchin, clinicians have increasingly viewed eating disorders not solely as intrapsychic disturbances but as relational phenomena embedded within family systems. These ideas led to structured, evidence-based models such as Family-Based Treatment (FBT) and Multifamily Therapy (MFT). This review summarizes historical milestones, theoretical innovations, and empirical findings on family-oriented interventions for eating disorders. The article also discusses mechanisms of change, clinical applications, and contemporary challenges in implementation and cultural adaptation of family based treatments. Some interesting research hypotheses are formulated regarding family support and neural circuitry during refeeding that can inspire future research.

## 1 Introduction

Eating disorders (EDs) such as anorexia nervosa (AN) and bulimia nervosa (BN) are complex biopsychosocial conditions with high morbidity and mortality (1). Early approaches emphasized intrapsychic conflict or biological predisposition, often focusing treatment on the individual. From the 1970s onward, systemic and family theorists reframed EDs as disorders of relational interaction, leading to a fundamental paradigm shift (2, 3).

The family-oriented movement in eating-disorder care arose simultaneously in Europe and North America, blending general systems theory, structural family therapy, and psychodynamic perspectives. It sought to understand how family patterns sustain or alleviate disordered eating and to harness family strengths for recovery. This review traces that evolution from early systemic formulations to current evidence-based models, highlighting research, practice, and future hypothetical directions.

It is however important to remark that in contemporary clinical practice, family-oriented therapy is best understood as one component within a multimodal treatment framework for eating disorders. Evidence-based care typically integrates nutritional rehabilitation, medical monitoring, and psychological interventions, including cognitive-behavioral approaches (e.g., CBT or CBT-E), with pharmacological support and inpatient or day-treatment programs when clinically indicated. Family-based and systemic interventions do not replace these modalities but rather complement them by mobilizing the family environment to support behavioral change, medical stabilization, and developmental recovery, particularly in children and adolescents. The present review focuses specifically on family-oriented approaches while acknowledging their role as part of an integrated continuum of care.

## 2 Review methodology

This article is conceived as a narrative, state-of-the-art review of family-oriented therapeutic approaches in the treatment of eating disorders. Rather than aiming for exhaustive systematic coverage, the review synthesizes historically influential models, landmark empirical studies, and contemporary developments that have shaped clinical practice and research over the past five decades.

The literature was identified through the author's longstanding engagement with the field and through targeted searches of major bibliographic databases commonly used in psychiatric and psychological research, including PubMed/MEDLINE, PsycINFO, and Web of Science. Searches focused on key terms such as *eating disorders*, *family therapy*, *family-based treatment*, *multifamily therapy*, *anorexia nervosa*, and *bulimia nervosa*, in various combinations.

The temporal scope spans from the early systemic formulations of the 1970s to recent empirical and implementation studies published up to 2025. Inclusion was guided by conceptual relevance, clinical influence, and empirical significance, with particular attention to foundational theoretical contributions, randomized controlled trials, meta-analyses, and major guideline-defining publications. The review prioritizes work that has had demonstrable impact on clinical models, treatment dissemination, or outcome research in child and adolescent populations.

## 3 Historical context and conceptual models

### 3.1 From individual pathology to relational systems

Before the 1970s, psychoanalytic and biological paradigms dominated conceptualizations of AN and BN (4). Hilde Bruch's seminal work *Eating Disorders: Obesity, Anorexia Nervosa, and the Person Within* (3) described deficits in autonomy and identity but retained an individual lens. Systems theorists such as Bateson (2) challenged this focus, proposing that communication within families forms a self-regulating network; symptoms serve to maintain equilibrium. An eating disorder thus becomes a communicative signal within an interactional field rather than an isolated pathology.

### 3.2 Mara selvini palazzoli and the Milan school

In Italy, psychiatrist Mara Selvini Palazzoli transitioned from psychoanalysis to family systems after observing the limited success of individual treatment for AN. In 1971 she established the Milan Center for Family Studies with Luigi Boscolo, Gianfranco Cecchin, and Giuliana Prata (4). Their “Milan model” emphasized circular questioning, hypothesizing, neutrality, and positive connotation. Symptoms were understood as relational communications preserving homeostasis in multigenerational networks. The Milan team's six-stage model of the anorectic process described how control, denial, and parental collusion maintain illness (5).

Therapy involved a team behind a one-way mirror, reflecting hypotheses and interventions to the family. By reframing the illness positively—e.g., “the symptom protects family unity”—the Milan group invited new interpretations and flexibility. Though primarily conceptual, the model influenced virtually all subsequent systemic treatments for EDs.

### 3.3 Salvador minuchin and the structural model

In Philadelphia, Salvador Minuchin developed Structural Family Therapy (SFT), emphasizing family organization, boundaries, and hierarchies (6). Studying “psychosomatic families”, Minuchin identified enmeshment, rigidity, over-protectiveness, and conflict avoidance as typical patterns surrounding AN (7). The symptom diverted attention from unresolved parental conflict or adolescent autonomy struggles. Treatment aimed to restructure boundaries through enactments and boundary-making exercises.

While later critics warned that Minuchin's language risked parent-blaming (8), his model provided observable criteria for change and a directive therapeutic stance. It operationalized systems theory for clinical practice and paved the way for later manualized models.

### 3.4 European synthesis and pragmatic family approaches

During the 1980s, European clinicians combined Milan systemic ideas with behavioral and psychodynamic approaches. Vandereycken, Kog, and Vanderlinden's volume “The Family Approach to Eating Disorders” (8) presented a pragmatic, non-blaming framework emphasizing assessment, conjoint sessions, and collaborative alliances. Families were reframed as resources rather than causes of illness. This work bridged conceptual sophistication with clinical practicality and influenced subsequent guideline development.

By the 1990s, controlled studies showed that involving families improved outcomes compared with individual therapy in adolescents with AN (9, 10). These findings laid the empirical groundwork for Family-Based Treatment (FBT).

## 4 Family-based treatment (FBT): from observation to evidence

In the late 1970s and early 1980s, clinicians at the Maudsley Hospital in London, including Christopher Dare, Ivan Eisler, Gerald Russell, and colleagues, translated systemic and structural concepts into a phased, manualized treatment designed specifically for adolescent anorexia nervosa (AN). This approach, first described by Russell and collegues (9), became known as Family-Based Treatment (FBT) or the Maudsley method. Although the Maudsley method is often presented as a discrete and well-defined treatment model, it is more accurately understood as a foundational framework that has continued to evolve in response to clinical experience, empirical findings, and broader developments in family therapy. Subsequent family-based interventions have extended the Maudsley approach both theoretically and technically, while retaining its core emphasis on parental empowerment and behavioral change.

FBT conceptualizes anorexia as a “biopsychosocial disorder” in which parents are the primary resource for facilitating recovery. The model is explicitly agnostic about etiology, assuming that family members did not cause the disorder but are best positioned to support the adolescent's restoration of healthy eating (15). FBT comprises three sequential phases:

Phase I: Weight restoration. Parents take full responsibility for supervising meals and interrupting weight-loss behaviors.
Phase II: Gradual return of control. As weight normalizes, control over eating is carefully transferred back to the adolescent.
Phase III: Adolescent development. Once healthy weight and eating patterns are sustained, sessions focus on normative autonomy and family relationships.

Key principles—sometimes called the five tenets of FBT—include parental empowerment, externalization of the illness, emphasis on early weight restoration, non-blaming stance, and pragmatic focus on behevior.

The evolution of family-based interventions following the Maudsley method can be broadly characterized along three intersecting dimensions. Theoretically, later models have incorporated insights from attachment theory, emotion regulation, and developmental psychopathology, thereby expanding the original agnostic stance toward a more nuanced understanding of relational and affective processes. Technically, adaptations have refined the use of parental coaching, meal support, and externalization strategies, and have introduced greater flexibility in session structure, therapist stance, and pacing of responsibility transfer. At the level of service delivery, family-based treatment has been extended into multifamily formats (see paragraph 5), parent-focused variants, and stepped-care and telehealth models, reflecting efforts to enhance engagement, acceptability, and scalability within diverse health-care system savior (11).

## 5 Empirical evidence and mechanisms of change

### 5.1 Controlled outcome research

Firstly, it should be noted however, that despite the overall consistency of findings favoring family-based interventions, the empirical literature is characterized by substantial heterogeneity in study design and outcome definition. Trials differ in age ranges, diagnostic thresholds, comparison conditions, and primary endpoints—most commonly weight restoration, remission status, or symptom reduction—as well as in follow-up duration. These methodological variations limit direct cross-study comparability and preclude simple aggregation of outcomes, underscoring the importance of cautious interpretation when synthesizing results across trials and reviews. Secondly, before summarizing empirical outcomes, it is important to clarify that the evidence supporting family-oriented interventions in eating disorders pertains to distinct treatment models and structures that have evolved over time. Early randomized controlled trials from the 1990s primarily evaluated forms of family therapy closely aligned with the original Maudsley method, whereas later studies increasingly examined manualized Family-Based Treatment (FBT), Multifamily Therapy (MFT), and a range of diagnostic and delivery adaptations. The empirical literature therefore reflects a developmental sequence of models, rather than a single, uniform family therapy approach.

The first randomized trial (9) compared family therapy to individual supportive counseling, showing superior weight recovery and menstruation resumption in family-treated adolescents. Replications confirmed these benefits, especially in younger patients and those with shorter illness duration (10, 12).

From the mid-1990s onward, randomized controlled trials (RCTs) became the foundation of empirical validation for family-oriented therapy in eating disorders. Early British and U.S. trials demonstrated that structured family interventions outperformed individual psychotherapy or eclectic approaches for adolescent anorexia nervosa (12–18). Across studies, approximately 45–50 percent of adolescents achieved full remission and up to 80 percent reached medically healthy weight within twelve months (19).

A meta-analysis by Couturier and colleagues (17) and later reviews (11, 16, 20) confirmed that family therapy significantly improves weight restoration, reduces relapse, and enhances long-term psychosocial functioning compared with non-family treatments. The 2018 Cochrane review (21) concluded that family therapy provides superior short-term weight outcomes and symptom reduction for adolescent anorexia nervosa, although heterogeneity of study designs limits precision of pooled effect sizes.

### 5.2 Extensions to other diagnoses

FBT was also applied to patients with bulimia nervosa (BN) (14, 22). Research on FBT for bulimia nervosa (FBT-BN) indicates moderate to large short-term advantages over individual cognitive-behavioral therapy (CBT) for adolescents (14). Abstinence from bingeing and purging occurred in 39–44 percent of FBT-BN participants at end of treatment vs. 20%–25% in CBT-A groups (14). At one-year follow-up, differences narrowed, yet FBT retained higher parental engagement and adherence rates. However, FBT-BN is best understood as a promising, developmentally informed adaptation rather than a fully equivalent evidence standard. CBT-E still remains the most evidence based treatment in the case of BN.

In the case of avoidant/restrictive food intake disorder (ARFID), family-oriented approaches are at an earlier stage of clinical and empirical development. Initial case series and pilot studies suggest that adapting family-based principles to ARFID—particularly parental support for exposure to feared foods and reduction of avoidance—can facilitate nutritional rehabilitation and functional improvement (19). Nevertheless, controlled trials remain limited, and the heterogeneity of ARFID presentations poses challenges for standardization. Current family-based interventions for ARFID should therefore be regarded as emerging models grounded in clinical rationale and preliminary evidence rather than established, evidence-based protocols.

### 5.3 Process and mediator studies

Beyond efficacy, research has explored mechanisms of change in family-based approaches. Early weight gain during the first four weeks of FBT strongly predicts full remission and sustained recovery (18, 23). Mediational analyses identify parental self-efficacy, reduced expressed emotion, and enhanced family cohesion as central drivers of outcome (24–26). Families characterized by lower criticism, shorter illness duration, and a united parental alliance respond most favorably (24–26).

Conversely, high parental psychopathology or intrafamilial conflict can impede progress; such families may benefit from parent-only or multifamily formats (15, 18, 26). The therapist's stance of neutrality and empowerment appears critical to maintaining engagement while preventing blame (7–9).

## 6 Multifamily therapy

### 6.1 Concept and clinical format

One of the latest evolutions in FBT (since 2010) is Multifamily Therapy (MFT) developed by Eisler and Asen at the Maudsley Hospital to intensify systemic work and combat isolation among families coping with eating disorders (27). Several families participate together in group sessions combining psychoeducation, role-plays, and reflective dialogue. Families witness parallels in others' struggles, which fosters normalization and collective problem-solving.

### 6.2 Empirical support

A pragmatic multicenter RCT (12) demonstrated comparable weight outcomes between MFT and single-family FBT but superior improvements in communication, general family functioning, and treatment satisfaction. Subsequent observational studies found that MFT benefits families with high expressed emotion or chronic illness (24, 25, 27). Hybrid models integrating MFT with parent-only sessions have further improved feasibility in outpatient services (20).

## 7 Clinical applications and therapist stance

### 7.1 Assessment and engagement

Family-oriented interventions begin with a detailed assessment of family organization, motivation, and medical risk. The therapist clarifies that parents are not blamed for the disorder but are essential partners in recovery. Psychoeducation reframes the eating disorder as an external, shared enemy. Early sessions often include a coached family meal, enabling the therapist to observe dynamics and coach supportive feeding behaviors (15).

### 7.2 Therapeutic principles

Core therapeutic strategies include externalization of the illness, reinforcement of parental unity, and gradual return of autonomy to the adolescent (15, 27). The therapist adopts a stance of firm empathy—directive in behavioral management yet respectful of developmental needs. Circular questioning and reframing convert blame into curiosity, transforming rigid family patterns into adaptive collaboration (7).

### 7.3 Common challenges

Therapists frequently encounter ambivalence, resistance, or high anxiety among parents and adolescents. Addressing these challenges involves validating emotional distress while maintaining behavioral expectations. Parental burnout, sibling resentment, and marital tension may require temporary subsystem sessions or liaison with supportive services. For families experiencing persistent hostility or psychiatric comorbidity, adjunctive parent-focused treatment (8) or multifamily formats (27) can enhance outcomes.

### 7.4 Termination and relapse prevention

In the final phase, therapy focuses on consolidating gains and developing relapse-prevention plans. Families identify early warning signs, reinforce coping strategies, and schedule booster sessions at three- and six-month intervals. The emphasis shifts from eating-behavior monitoring to communication, autonomy, and identity development—markers of sustainable recovery (15, 17, 27).

## 8 Integration with health-service systems

### 8.1 Policy and implementation

The endorsement of family therapy in the United Kingdom's NICE Guidelines (28) and the NHS England Access and Waiting Time Standard (29) institutionalized systemic treatment in national services. Community-based FBT has reduced hospital admissions and shortened inpatient stays, generating substantial cost savings (17, 20). In many countries, stepped-care frameworks prioritize outpatient FBT for medically stable adolescents, reserving hospitalization for acute cases (26, 30).

### 8.2 Training and dissemination

Large-scale implementation requires formal training and supervision to ensure fidelity. Studies show that with structured supervision, community clinicians can deliver FBT with comparable effectiveness to academic centers (30). Recent telehealth adaptations (31, 32) and online coaching programs extend family-based care to underserved regions without loss of efficacy.

Digital innovation is reshaping the field. Tele-FBT and blended online programs have expanded access while maintaining clinical fidelity (32). Web-based psychoeducation platforms help parents learn refeeding skills and track progress between sessions. Early outcome studies show comparable results to in-person treatment when therapist contact and monitoring remain consistent (31).

## 9 Hypotheses and research directions on family support and neural circuitry during refeeding

The following section adopts a theoretical and hypothesis-generating perspective to explore potential links between family support, refeeding processes, and neural circuitry in eating disorders. While emerging findings from neuroscience and psychophysiology provide suggestive correlates, these formulations should be understood as conceptual integrations rather than empirically established mechanisms.

Neuroimaging studies have demonstrated alterations in reward and anxiety circuits among individuals with anorexia nervosa and related conditions, notably within dopaminergic and limbic pathways that influence motivation and emotional regulation (33, 34). However, little is known about how social support—especially from family members—modulates these neural systems in real time. Theoretical frameworks from interpersonal neurobiology and attachment theory propose that relational attunement and co-regulation foster neurophysiological states of safety, potentially influencing the functioning of reward-related and stress-responsive circuits (35–37). Empirical evidence also underscores the importance of family functioning and perceived relational quality in treatment outcomes for eating disorders (38). Therefore, a promising avenue for future research is to investigate whether supportive, emotionally attuned family interactions during refeeding (42) are associated with measurable changes in neural activity within reward-related regions such as the ventral striatum and orbitofrontal cortex, and anxiety-related regions such as the amygdala and insula. Such studies could bridge the gap between psychosocial and neurobiological models of eating disorders, offering novel insight into how interpersonal processes contribute to recovery and resilience at the neural level. Future research may for instance discover that successful family therapy entails embodied as well as cognitive change, families may “learn” new physiological patterns of connection that reinforce recovery. At present, these proposed links between family processes and neural regulation remain largely untested at a mechanistic level and should be regarded as directions for future interdisciplinary research, rather than as explanatory models supported by direct causal evidence.

## 10 Strengths, challenges, and ongoing debates

Family-oriented therapy has transformed ED treatment by redefining parents as therapeutic partners rather than sources of pathology. Its strengths include clear behavioral focus, replicability, and congruence with developmental theory. Moreover, FBT and MFT achieve durable outcomes across varied health systems and cost-effective scalability through manualization (17, 27, 43).

Nonetheless, challenges persist. Conclusions regarding treatment “superiority” in this literature should be understood as referring primarily to clinical outcomes rather than to comparative acceptability or family experience, which remain less consistently evaluated across most studies. Accordingly, FBT's efficacy is strongest in adolescents with anorexia nervosa and with relatively short illness duration and intact family structures (26). Effectiveness for adults, chronic cases, or culturally diverse families is less established (39, 40). Some clinicians question whether FBT underemphasizes emotion processing or autonomy development, proposing integration with Emotion-Focused Family Therapy (41) or Cognitive-Behavioral Therapy–Enhanced (CBT-E) modules (44). Ongoing refinement aims to balance behavioral rigor with emotional depth and cultural sensitivity.

Cultural adaptation is a critical frontier. Trials in East Asia and Latin America emphasize collectivist values and multigenerational involvement, requiring adjustment of Western assumptions about parental authority and individuation (39). Translation and localization of manuals, supervision standards, and digital content remain priorities for global dissemination.

## 11 Future directions

Emerging work seeks to personalize family-based interventions through precision-medicine approaches. Predictive modeling may soon tailor treatment intensity to baseline risk profiles, parental psychopathology, and family dynamics (20). Integrative frameworks combining systemic, attachment, and neurobiological perspectives can illuminate how family interactions regulate stress physiology and reinforce recovery behaviors.

The next generation of research should emphasize longitudinal follow-up, mechanism testing, and health-economic outcomes. Training programs must include not only protocol adherence but also systemic conceptualization skills and sensitivity to cultural and developmental factors. Hybrid telehealth models promise to extend reach while maintaining therapeutic alliance (32). Digital innovation now shapes delivery. Tele-FBT and blended online formats preserve treatment fidelity while enhancing accessibility. Early data show non-inferiority to in-person FBT when structured supervision and monitoring are maintained. Such approaches are vital for reaching families distant from specialty centers. Ultimately, future family-oriented therapy will likely blend relational, technological, and biological knowledge into a unified, flexible system of care (38).

## 12 Conclusion

From the systemic formulations of Mara Selvini Palazzoli and Salvador Minuchin to contemporary evidence-based interventions such as FBT and MFT, family- oriented therapy has revolutionized the treatment of eating disorders, especially in the case of the treatment of adolescent anorexia nervosa. Extensions to other diagnoses such as BN, ARFID, adults with eating disorders and chronic eating disorders remain emerging and efficacy of family oriented treatment in these diagnostic groups needs more being researched in the nearby future. This means that transdiagnostic applications are promising but unevenly supported by evidence-based data.

Another important limitation of the present synthesis concerns the contextual concentration of the evidence base. The majority of randomized trials, implementation studies, and guideline-defining publications on family-based treatment originate from Western, high-income health systems and are embedded within cultural assumptions emphasizing nuclear-family structures, parental authority, and access to specialized outpatient services. As a result, the feasibility, acceptability, and effectiveness of these models may be moderated by cultural norms, family organization, and health-system infrastructure in non-Western or resource-limited settings. While emerging adaptations in diverse contexts are promising (39, 40), generalizability beyond the service environments in which these models were developed should be interpreted with appropriate caution

While family-oriented therapy is firmly supported by clinical and outcome research, ongoing advances in neuroscience and related fields may offer future opportunities to refine theoretical models of change. At present, such neurobiological perspectives remain complementary and exploratory, rather than constitutive of the evidence base supporting family-based interventions. Collaboration among researchers, clinicians, and families themselves will ensure that the field continues to evolve toward more inclusive, personalized, and effective models of recovery.
